# Engineering Microbial Consortia for High-Performance Cellulosic Hydrolyzates-Fed Microbial Fuel Cells

**DOI:** 10.3389/fmicb.2019.00409

**Published:** 2019-03-18

**Authors:** Feng Li, Xingjuan An, Deguang Wu, Jing Xu, Yuanyuan Chen, Wenchao Li, Yingxiu Cao, Xuewu Guo, Xue Lin, Congfa Li, Sixin Liu, Hao Song

**Affiliations:** ^1^Key Laboratory of Systems Bioengineering (MOE), Frontier Science Center for Synthetic Biology, School of Chemical Engineering and Technology, Tianjin University, Tianjin, China; ^2^Department of Brewing Engineering, Moutai Institute, Renhuai, China; ^3^Tianjin Engineering Research Center of Microbial Metabolism and Fermentation Process Control, Tianjin University of Science and Technology, Tianjin, China; ^4^College of Food Science and Technology, Hainan University, Haikou, China

**Keywords:** microbial fuel cell, synthetic biology, microbial consortia, cellulosic hydrolyzates, *Shewanella oneidensis*

## Abstract

Microbial fuel cells (MFCs) are eco-friendly bio-electrochemical reactors that use exoelectrogens as biocatalyst for electricity harvest from organic biomass, which could also be used as biosensors for long-term environmental monitoring. Glucose and xylose, as the primary ingredients from cellulose hydrolyzates, is an appealing substrate for MFC. Nevertheless, neither xylose nor glucose can be utilized as carbon source by well-studied exoelectrogens such as *Shewanella oneidensis*. In this study, to harvest the electricity by rapidly harnessing xylose and glucose from corn stalk hydrolysate, we herein firstly designed glucose and xylose co-fed engineered *Klebsiella pneumoniae-S. oneidensis* microbial consortium, in which *K. pneumoniae* as the fermenter converted glucose and xylose into lactate to feed the exoelectrogens (*S. oneidensis*). To produce more lactate in *K. pneumoniae*, we eliminated the ethanol and acetate pathway via deleting *pta* (phosphotransacetylase gene) and *adhE* (alcohol dehydrogenase gene) and further constructed a synthesis and delivery system through expressing *ldhD* (lactate dehydrogenase gene) and *lldP* (lactate transporter gene). To facilitate extracellular electron transfer (EET) of *S. oneidensis*, a biosynthetic flavins pathway from *Bacillus subtilis* was expressed in a highly hydrophobic *S. oneidensis* CP-S1, which not only improved direct-contacted EET via enhancing *S. oneidensis* adhesion to the carbon electrode but also accelerated the flavins-mediated EET via increasing flavins synthesis. Furthermore, we optimized the ratio of glucose and xylose concentration to provide a stable carbon source supply in MFCs for higher power density. The glucose and xylose co-fed MFC inoculated with the recombinant consortium generated a maximum power density of 104.7 ± 10.0 mW/m^2^, which was 7.2-folds higher than that of the wild-type consortium (12.7 ± 8.0 mW/m^2^). Lastly, we used this synthetic microbial consortium in the corn straw hydrolyzates-fed MFC, obtaining a power density 23.5 ± 6.0 mW/m^2^.

## Introduction

Microbial electrochemical technologies are green and sustainable that enabled many practical applications in environments and energy fields (Wang and Ren, 2013; Wang et al., 2015), including microbial fuel cells (MFCs) to harvest electricity production from organic wastes treatment (Bond et al., 2002; Lovley, 2006; Logan, 2009; Logan and Rabaey, 2012), microbial electrolysis cells to product hydrogen (Cheng and Logan, 2007; Kadier et al., 2016), microbial electrosynthesis to produce value-added chemicals and biofuels from CO_2_ bio-reduction (Rabaey and Rozendal, 2010; Li et al., 2012; Liu et al., 2016), and MFC-based biosensors for remote environmental monitoring in long-term (Golitsch et al., 2013), and so on. Meanwhile, MFCs are interesting devices to study interspecies extracellular electron transfer in mixed microbial consortia (Katoa et al., 2012; Ha et al., 2017). A large amount of efforts had been made to design MFCs that could harvest electricity from brewery wastewaters (Wen et al., 2010), human feces (Du et al., 2011), marine putrefaction (Tender et al., 2002), natural gas (McAnulty et al., 2017; Yamasaki et al., 2018), and lignocellulose biomass (Mohan et al., 2016), etc. Cellulose is the most abundant biomass in the world, the hydrolyzates of which are rich in glucose and xylose (Payne et al., 2015; Kracher et al., 2016; Taylor et al., 2018). Thus, the conversion of xylose and glucose to electricity energy by designing novel MFCs has received much attention in recent years (Yang et al., 2015b; Lin et al., 2016; Li et al., 2017; Liu et al., 2017).

*Shewanella oneidensis*, one of the most well-studied exoelectrogens (Hau and Gralnick, 2007; Fredrickson et al., 2008; Li et al., 2018a,c), is capable of conducting extracellular electrons transfer (EET) to anodes via direct electron transfer pathways mediated by *c*-type cytochromes, and electron transfer pathways mediated by diffusible electron shuttles (Shi et al., 2016; Kumar et al., 2017). Nevertheless, the wild-type (WT) *S. oneidensis* could only utilize very limited substrates (i.e., acetate, lactate, and pyruvate) as their carbon and energy sources, while common pentoses or hexoses from cellulose could not be utilized by the WT *S. oneidensis* due to its incomplete sugar metabolism (Pinchuk et al., 2009; Flynn et al., 2012), which enormously restricted the wide applications of *S. oneidensis*. To use xylose and glucose as substrates in MFC for harvesting electricity, a number of synthetic biology and microbial consortium strategies were developed. Li et al. successfully constructed engineered *S. oneidensis* by assembling the xylose transporters from *Candida intermedia* with one of intracellular oxidoreductase pathway from *Schefersomyces stipites* (Li et al., 2017). Furthermore, the glucose metabolic pathways from *Zymomonas mobilis* were heterogeneously expressed into *S. oneidensis*, allowing it to use glucose as the sole carbon and energy source (Choi et al., 2014). However, these efforts only used either glucose or xylose for harvesting energy, not achieving co-utilization of pentoses and hexoses from cellulose hydrolyzates for electricity production. Another efficient strategy was to employ mixed microbial cultures to derive electricity from cellulose hydrolyzates in MFCs. Undefined mixed electricity-producing microbial communities in MFCs with various biomass hydrolysates including wheat straw hydrolysate (Zhang et al., 2009), rapeseed straw hydrolysate (Jablonska et al., 2016), rice straw hydrolysate (Wang et al., 2014) were used for electricity generation. However, it is hard to further improve the power generation of such undefined microbial mixture due to the complicated mechanisms of the microbial metabolic interactions and ambiguous EET among fermenters and exoelectrogens. However, recent studies demonstrated that *K. pneumonia*, one of the most well-established model microorganisms (Kumar and Park, 2017), was capable of utilizing a wide spectrum of substrates as carbon sources (such as xylose, glucose, and glycerol, etc.) to synthesize lactate (Feng et al., 2014). Also, *K. pneumonia* exhibited high growth rate, which endowed it more metabolic advantages over other fermentative strains used in electrochemical systems. Furthermore, biotechnological applications of *K. pneumonia* will also require attenuation of its pathogenicity and reduction of biosafety concerns. Many virulence factors that contribute to its pathogenicity including lipopolysaccharide (LPS), capsular antigens, fimbrial adhesins, siderophores, and O antigens, have been inactivated by genetically engineering approaches (Kumar and Park, 2017).

**Figure 1:**
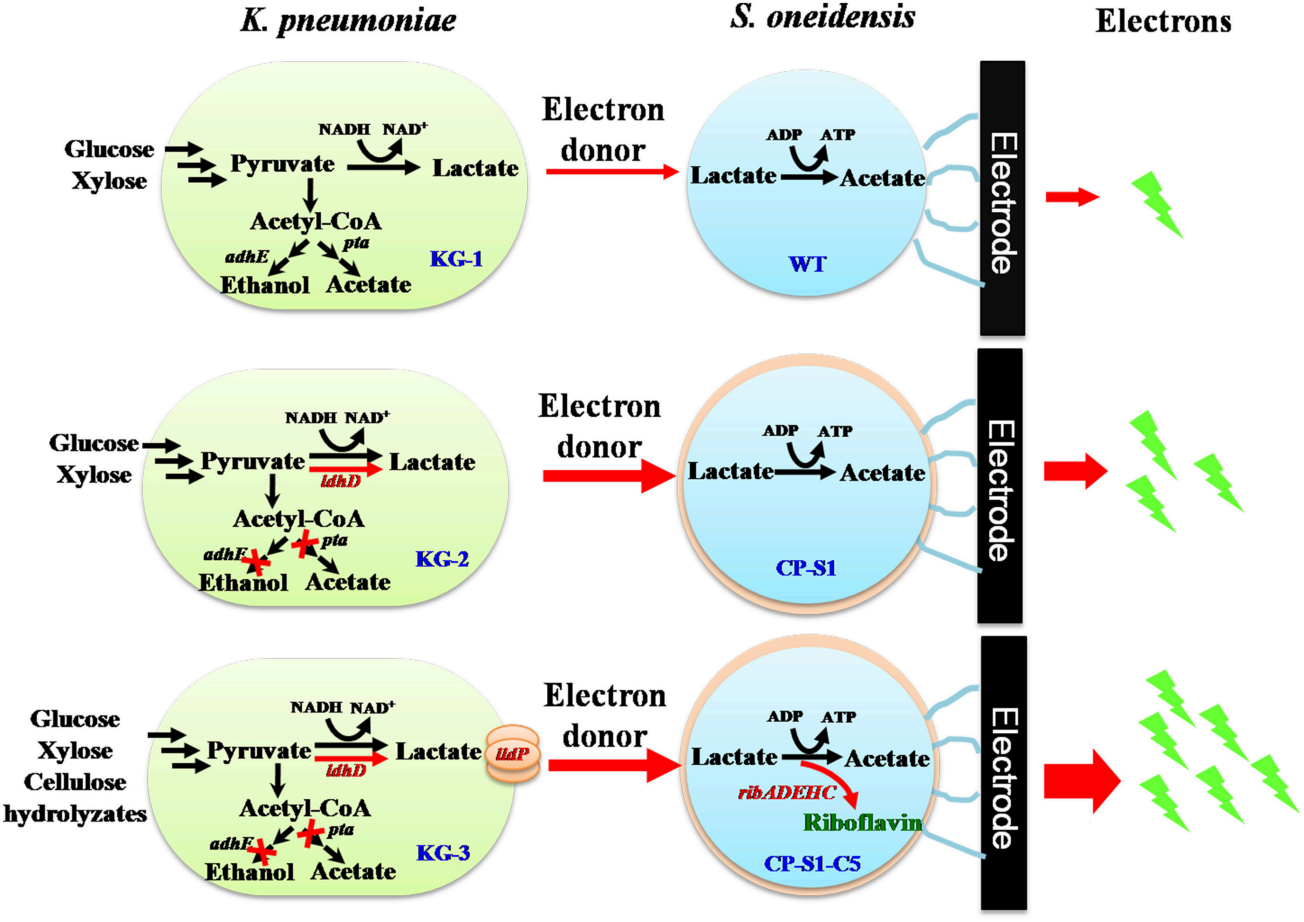
Schematic of the metabolic interaction of the engineered microbial consortia with *K. pneumoniae S. oneidensis* in a hierarchical way. Lactate was produced by *K. pneumoniae* from glucose and xylose which was fed to *S. oneidensis* as carbon source and electron donor to generate electricity in MFCs. To direct more carbon flux to lactate biosynthesis of *K. pneumoniae*, we eliminated the ethanol and acetate pathway via deleting phosphotransacetylase gene (*pta*) and alcohol dehydrogenase gene (*adhE*) and further constructed a synthesis and delivery system through expressing a lactate dehydrogenase gene *(ldhD)* and a lactate transporter gene (*lldP*). To increase *S. oneidensis* adhesion to the carbon electrode to compete the surface of electrode and further facilitate flavin-mediated electron transfer, a biosynthetic flavins pathway (from *Bacillus subtilis*) was expressed in a highly hydrophobic *S. oneidensis* strain CP-S1.

To further promote bioelectricity generation efficiency from cellulose hydrolyzates, we herein designed a rationally engineered microbial consortium based on the principle of “division-of-labor” (Liu et al., 2017), in which metabolic interaction modes between the fermenter (*K. pneumoniae*) and the exoelectrogen (*S. oneidensis*) were increased to achieve an efficient conversion of cellulose hydrolyzates to bioelectricity energy. As shown in Figure 1, we rationally engineered the consortium strains by redirecting carbon flux distribution of the *K. pneumoniae* and improving extracellular electron transfer of the *S. oneidensis*, respectively. To promote more carbon flux to lactate biosynthesis of *K. pneumoniae*, we firstly knocked out the phosphotransacetylase gene (*pta*) after deleting the alcohol dehydrogenase gene (*adhE*) to simultaneously eliminate the ethanol and acetate pathway (Guo et al., 2014). Then, a synthesis and delivery system, including a lactate dehydrogenase encoded by *ldhD* gene from *Lactobacillus bulgaricus* and a lactate transporter encoded by *lldP* gene from *Escherichia coli* (Li et al., 2015), was expressed to accelerate the supply of lactate. To improve the electrons transfer of *S. oneidensis*, a biosynthetic flavins pathway from *Bacillus subtilis* was expressed in a highly hydrophobic *S. oneidensis* strain CP-S1, which not only improved direct-contacted EET via enhancing *S. oneidensis* adhesion to the carbon electrode but also accelerated the flavins-mediated EET via increasing flavins synthesis. As a result, the mixed sugars (glucose and xylose) and cellulose hydrolyzates-fed MFC, inoculated with the engineered *K. pneumonia-S. oneidensis*, produced a maximum power density of 104.7 ± 10.0 mW/m^2^ and 23.5 ± 6.0 mW/m^2^. This synthetic microbial consortium successfully utilized cellulose hydrolyzates to harvest electricity, laying the foundation for the efficient conversion of biomass to generate power in MFCs.

## Materials and Methods

### *In vitro* Gene Synthesis

The gene sequences of *ldhD* from *L. bulgaricus* ATCC11842 and the *lldP* from *E. coli* K-12 strain MG1655 were extracted from the NCBI database (www.ncbi.nlm.nih.gov/gene/) (Table S1). To prevent shortage of tRNAs for rare codons, we used a Java codon adaption tool (JCAT) to obtain the optimal sequences for *K.pneumoniae* (Yang et al., 2015a). The *ldhD* gene and *lldP* gene were then *in vitro* synthesized. Subsequently, the sequences of *ldhD* and *lldP* were linked in vector pUC18K, obtaining recombinant plasmid pUC18K-ldhD-lldP. The designed gene sequences were synthesized and verified *in vitro*. All the plasmid construction was performed in *E. coli* Trans T1.

### Strain Construction and Culture

The KG-2 strain was obtained by deleting the *adhE* gene and the *pta* gene from KG-1 by following a previously established procedure (Guo et al., 2014). The recombinant *K. pneumoniae* strain KG-3 was engineered by electro-transferring the plasmid pUC18K-ldhD-lldP into KG-2. Two millimeter electroporate chamber and pulser voltage selected 1.8 kV were used in this electro-transferring process. The plasmid pYYDT-C5 harboring the flavin synthesis genes (*ribADEHC*) from *B. subtilis* (Yang et al., 2015a) was transformed into *E. coli* WM3064, and then was transferred into a highly hydrophobic *S. oneidensis* strain CP-S1 by conjugation. All stains and plasmids used in this study are listed in Table 1.

**Table 1:** Strains and plasmids used in this study.

**Strains or plasmids**	**Feature(s)**	**Source**
Strains		
***K. PNEUMONIAE***		
KG-1(WT *_*K*.*pneumoniae*_*)	Wild type *K.pneumoniae*	Our lab
KG-2	KG1*ΔadhEΔpta*	This study
KG-3	Carrying plasmid pUC18K- *ldhD-lldP*	This study
***S. ONEIDENSIS***		
MR-1(WT *_*S*.*oneidensis*_*)	Wild type *S. oneidensis*	Our lab
CP-S1	MR-1*Δ-1S1*	Our lab
CP-S1-C5	Carrying pYYDT-C5	Our lab
***E. COLI***		
TransT1	F-80(lacZ)ΔM15ΔlacX74hsdR(rk-, mk+)ΔrecA1398endAltonA	
WM3064	A dap auxtotroph *E.coli*	Our lab
**PLASMIDS**		
pUC18k	Kam^r^, pUC ori, P_tac_, MCS	Our lab
pUC18k-*ldhD-lldP*	Plasmid with *ldhP* and *lldP* gene inserted	This study
pYYDT	5.9 kb,Km^r^,*lacZ*	Our lab
pYYDT-C5	Plasmid with the *ribA,ribD,ribE,ribH* and *ribE* gene inserted	Our lab

### MFC Setup

The H-type MFCs were separated by Nafion 117 membranes, which were pretreated as those used in the previous study. The anode and cathode were composed of carbon cloth for anode (2.5 cm × 2.5 cm) and cathode (2.5 cm × 3 cm). The anodic electrolyte consisted of M9 buffer (Table S2) supplemented with definite carbon source and 5% (v/v) LB broth. The cathodic electrolyte was made of 50 mM KH_2_PO_4_, 50 mM K_2_HPO_4_, and 50 mM K_3_[Fe(CN)_6_] solution. The amplification cultured cells (OD600 = 2.0) were centrifuged, collected, and dispersed the anode chambers. To ensure consistent culture condition for different MFCs, 50 μg/mL kanamycin and 0.2 mM IPTG were supplemented in anodic electrolyte. All the MFCs were incubated at 30°C, and the voltage generation was measured by a digital multimeter (DT9205A) across the external loading a 2 kΩ resistor in MFCs.

### Quantification of Metabolites

The metabolites in the shake flasks were analyzed using a high performance liquid chromatography (HPLC) system equipped with a refractive index detector. All samples and standard solutions were diluted and filtered by a 0.22 μm filter prior to HPLC assay. To analyses glucose, xylose, lactate, acetate, and ethanol, 5 mM sulfuric acid was used as the mobile phase (0.6 mL/min) through the Aminex HPX-87H ion-exchange column (Bio-Rad) which was incubated at 45°C, and detection was performed with the Waters 2414 refractive index detector. In addition, the determination of riboflavin concentration can refer to our previous method (Li et al., 2018a).

### Bio-Electrochemical Analysis

Electrochemical analyses were performed on a CHI 1000C multichannel potentiostat (CH Instrument, Shanghai, China), when MFCs reached the pseudo-steady state. Cyclic voltammetry (CV) was conducted on a three-electrode configuration with the cathode as the counter electrode, the anode as the working electrode, and the Ag/AgCl (vs. SHE) as the reference electrode. To obtain the maximum power density and the polarization curves, linear sweep voltammetry (LSV) analysis was also performed on the CHI 1000C multichannel potentiostat.

The actually recovered coulombs were determined by integrating the current (*I*) over a period of batch cycle (*t*_*b*_). Such that the coulombic efficiency can be evaluated as Yan et al. (2015); Li et al. (2018a,c):

$$\begin{array}{l} C_{E}=\frac{Coulombs\;recovered}{Total\;coulombs\;in\;substrate}=\frac{M_{S}\overset{t_{b}}{\underset{0}{\int }}Idt}{Fb_{ES}V_{An}\Delta c}=\frac{M_{S}It_{b}}{Fb_{ES}V_{An}\Delta c} \end{array}$$

where *M*_*S*_ (g/mol) is the molecular weight of the substrate, *F* is the Faraday's constant (98.485 C/mol of electrons), *C* is the symbol of coulomb, *I* (A) is the current, *t*_*b*_ (s) is the time period of a batch cycle, *b*_*ES*_ is the stoichiometric number of moles of electrons produced per mole of substrate (glucose: *b*_*ES*_ = 16 when acetate was used; xylose: *b*_*ES*_ = 12 when acetate was used), *V*_*An*_ (L) is the volume of liquid in the anode compartment, and Δ*c* (g/L) is the change in substrate concentration over a bath cycle time.

### Biofilms Characterization

To measure the composition of microbial consortium, anode carbon were sampled and assayed by colony forming unit (CFU) counting. The anode was placed in a 50 mL test tube containing 5 mL of PBS (pH = 7.2) solution, vortexed oscillation for 2 min. A series of dilutions were spread onto LB agar plates, and incubated at 30°C for 36 h. The colony of *S. oneidensis* and *K. pneumoniae* was dark orange and white, respectively.

### Preparation of Corn Straw Hydrolysates

Corn straw (CS) used in this study was harvested in the suburb of Tianjin, China. CS was milled coarsely using a beater pulverizer and the fractions between 20 and 80 meshes were screened. The CS was further transformed to hydrolysates upon pretreatment and enzymatic hydrolysis. The procedure of pretreatment was carried out by following a previously published procedure with minor modifications (Li et al., 2016, 2018c). And the enzymatic hydrolysis experiments were performed in accordance with the NREL standard protocol (LAP-009). After enzymatic hydrolysis at 30°C and 200 rpm for 72 h in an orbital incubator, the mixture was centrifuged at 12,000 rpm for 5 min to separate the hydrolysates from solid residues. The hydrolysates were frozen at −20°C for subsequent sugar analysis.

## Results and Discussion

### Increase Lactate Production From *K. pneumoniae* and Strengthen Flavins Biosynthesis From *S. oneidensis*

**Figure 2:**
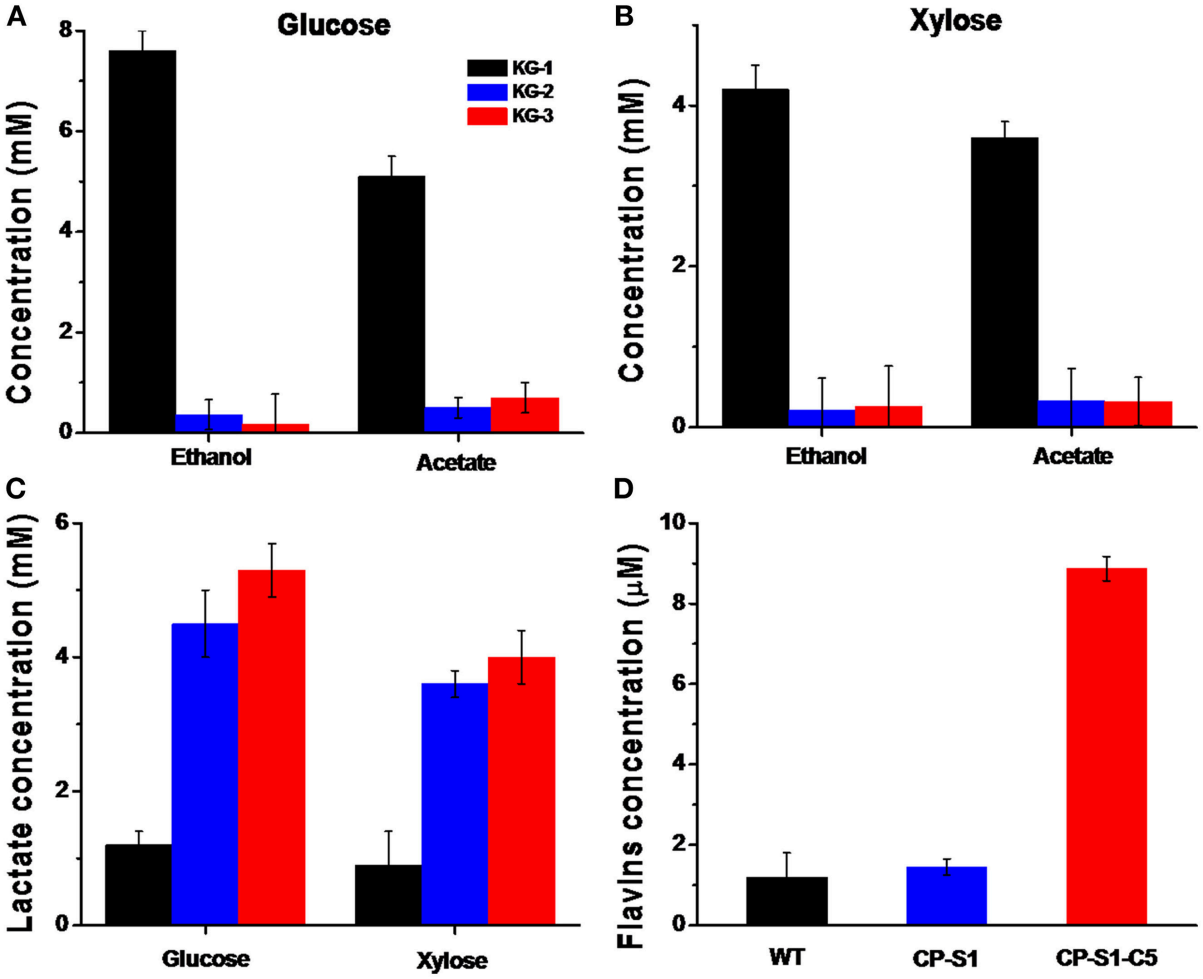
Quantification of metabolites of *K.pneumoniae* and *S.oneidensis*. The metabolites concentration in the fermentation media (*K. pneumoniae* was inoculated in the anolyte with 20 mM glucose or 20 mM xylose, and *S. oneidensis* was cultured with 20 mM of sodium L-lactate) was quantified by HPLC after 24 hours' fermentation, respectively. **(A)** Quantification of metabolites when *K. pneumoniae* was inoculated in the anolyte with 20 mM glucose. The concentration of lactate produced by KG-3 increased by 2.1 times than KG-1(from 2.8 ± 0.2 mM to 8.8 ± 0.4 mM); the ethanol level produced by KG-3 decreased by 97% (from 7.6 ± 0.5 mM to 0.2 ± 0.1 mM); and the acetate level produced by KG-3 decreased by 86% (from 5.1 ± 0.4 mM to 0.7 ± 0.3 mM). **(B)** Quantification of metabolites when *K. pneumoniae* was inoculated in the anolyte with 20 mM xylose. The concentration of lactate produced by KG-3 increased by 2.4 times than KG-1(from 1.8 ± 0.4 mM to 6.2 ± 0.5 mM); the ethanol level produced by KG-3 decreased by 94% than KG-1 (from 5.2 ± 0.3 mM to 0.3 ± 0.2 mM); the acetate level produced by KG-3 decreased by 81% (from 3.6 ± 0.2 mM to 0.7 ± 0.3 mM). **(C)** The concentration of lactate produced by KG-3 increased by 3.4 times than KG-1(from 1.2 ± 0.2 mM to 5.3 ± 0.4 mM) when inoculated in the anolyte with 20 mM glucose; The concentration of lactate produced by KG-3 increased by 3.4 times than KG-1 (from 0.9 ± 0.5 mM to 4.0 ± 0.5 mM) when inoculated in the anolyte with 20 mM xylose. **(D)** The concentration of flavins obtained from CP-S1-C5 increased ~6.4 times (from 1.2 ± 0.1 μM to 8.9 ± 0.3 μM) compared with the wild-type strain (MR-1). Statistics were calculated from three independent replicates of experiments.

As the fermenter, *K. pneumoniae* could utilize glucose or xylose as a carbon source to produce lactate, concurrently producing a considerable amount of by-products, i.e., ethanol and acetate (Guo et al., 2014). Therefore, to drive more glucose or xylose to synthesize lactate, we eliminated the ethanol and acetate biosynthesis pathways by knocking out the gene *adhE* encoding the alcohol dehydrogenase and gene *pta* encoding phosphotransacetylase in *K. pneumoniae*. However, interruption of ethanol biosynthesis would accumulate abundant intracellular reducing equivalent (NADH), causing unbalancing of intracellular redox, which further inhibited cell viability. To solve this redox unbalance and increase lactate synthesis, we expressed the *ldhD* gene encoding lactate dehydrogenase from *L. bulgaricus*, thus consuming the excess NADH. Concurrently, a lactate transporter encoded by *lldP* gene from *E. coli* was introduced in *K. pneumonia* to increase the lactate transportation across the hydrophobic cell membrane. Thus, as shown in Figures 2A–C, compared with the wild *K. pneumonia* KG-1, the recombinant *K. pneumoniae* strain KG-3 expressed lactate biosynthesis pathway, deleted by-products synthesis and produced a significant amount of lactate, almost in the absence of ethanol and acetate.

In addition, based on different electron transfer mechanisms, two strategies were adopted to facilitate extracellular electron transport. To improve the direct contact-based electron transfer pathway of exoelectrogens, the CP-S1 *S. oneidensis* mutant was used to further improve the adhesion of *S. oneidensis* to the carbon electrode surfaces (Figure S2). To enhance the shuttles-mediated electrons transfer, a flavin biosynthesis pathway was assembled in CP-S1 to further accelerate electron transfer. As a result, the flavins produced by the CP-S1-C5 increased by 6.4-folds compared with that of the WT *S. oneidensis* MR-1 (from 1.2 ± 0.1 μM to 8.9 ± 0.3 μM) (Figure 2D).

### Optimize *K. pneumonia-S. oneidensis* Microbial Consortium in the Mixed Sugar Co-fed MFC

**Figure 3:**
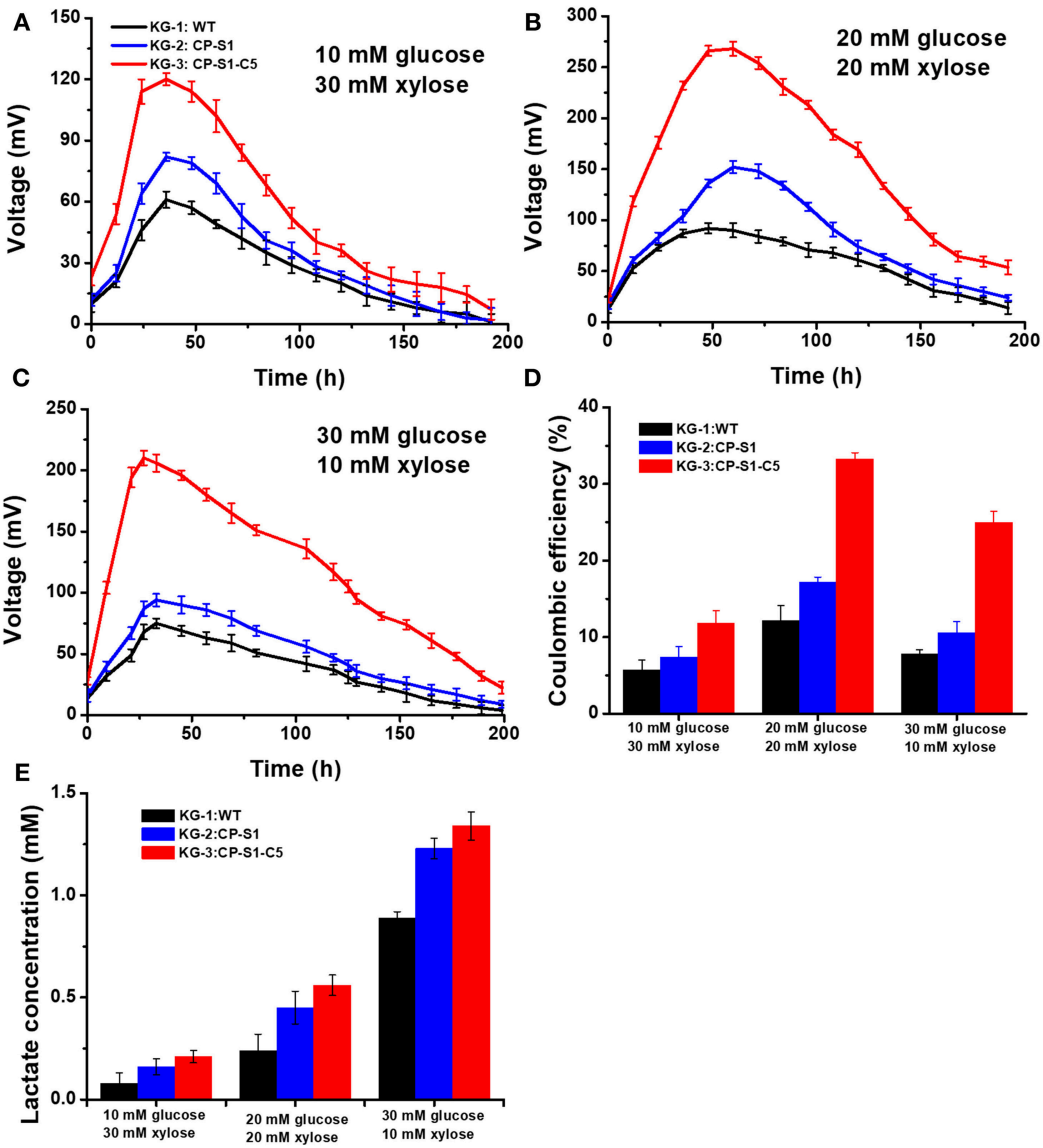
Optimizing glucose and xylose concentration of the *K.pneumoniae S.oneidensis* consortia to maximize power output in MFCs. The MFC voltage output of *K.pneumoniae S.oneidensis* consortia with different concentrations of feed-in glucose and xylose. **(A)** Corresponded to feed-in 10 mM glucose and 30 mM xylose. **(B)** Corresponded to feed-in 20 mM glucose and 20 mM xylose. **(C)** Corresponded to feed-in 30 mM glucose and 10 mM xylose. **(D)** The coulomb efficiency of the *K. pneumoniae S. oneidensis* consortia-inoculated MFCs with different feed-in glucose and xylose concentrations. **(E)** Analysis of lactate level at 24 th h under different feed-in glucose and xylose concentrations.

To realize a continuous and stable supply of carbon sources for exoelectrogen and achieve a maximum MFC power generation, we optimized the seeding ratio of the microorganisms and the feed-in glucose and xylose concentration, respectively. Firstly, the seeding ratio of the *K. pneumoniae* and *S. oneidensis* was optimized by a serial of orthogonal experiments to achieve a maximum MFC power generation in anolytes. The result indicated that the seeding OD_600_ ratio between *K. pneumoniae* and *S. oneidensis* being 1 to 5 would reach a maximum bioelectricity generation in MFCs (Figure S1). Secondly, to ensure that *S. oneidensis* had a suitable continuous supply of lactate produced by *K. pneumoniae* for the maximum MFC power generation, we further optimized the initial feed-in glucose and xylose concentrations. Three different concentration ratios of glucose and xylose, i.e., 30 mM (glucose): 10 mM (xylose), 20 mM: 20 mM, and 10 mM: 30mM xylose were chosen to determine the optimal substrate concentration for our engineered strains. As shown in Figures 3A–C, the results showed that the feed-in ratio of 20 mM (glucose): 20 mM (xylose) reached a highest output voltage (268.5 ± 10 mV), while 10 mM: 30 mM and 30 mM: 10 mM generated a lowest MFC output voltage, 120.2 ± 7.0 mV and 210.3 ± 6.0 mV, respectively. Furthermore, as shown in Figure 3D, the coulomb efficiency of the MFC inoculated with KG-3: CP-S1-C5 and substrates (glucose: xylose = 20 mM: 20 mM) was 33.3%, which was higher than in the other scenarios.

To illuminate the underlying reason, the lactate concentrations sampled from the anolyte were measured after 24 h cultivation. The data indicated that feed-in ratio 30 mM (glucose) and 10 mM (xylose) could cause a higher level accumulation of lactate in MFCs (Figure 3E), however, the MFC output voltage was not the optimum. This result was caused by the fact that the rapidly accumulated lactate resulted in an acidic condition (pH = 5), in which the growth activity and flavins biosynthesis of cell would be inhibited, reducing the power generation capability of *S. onedensis* (Yong et al., 2013; Lin et al., 2016). When feeding in 10 mM glucose and 30 mM xylose in MFC, the voltage output maintained at a low level all the time. This could be explained as the substrate could be exhausted rapidly, leading to a shortage of lactate to feed *S. oneidensis*. However, upon feeding 20 mM glucose and 20 mM xylose, the recombinant consortium enabled higher and longer voltage output than other concentrations. In this scenario, *K. pneumoniae* can continuously supply lactate to *S. oneidensis*, and the lactate from this feed-in mix sugar concentration was neither too high to generate cellular toxicity to *S. oneidensis*, nor too low as substrates to feed *S. oneidensis*. In addition, the substrates consumption results demonstrated that the engineered strain KG-3 showed a superior performance of substrate consumption than that of the KG-1 and the KG-2 (Table S3). Meanwhile, these strains preferred to utilize glucose than xylose for the production of lactate.

### MFC Performance and Bio-Electrochemical Analysis

**Figure 4:**
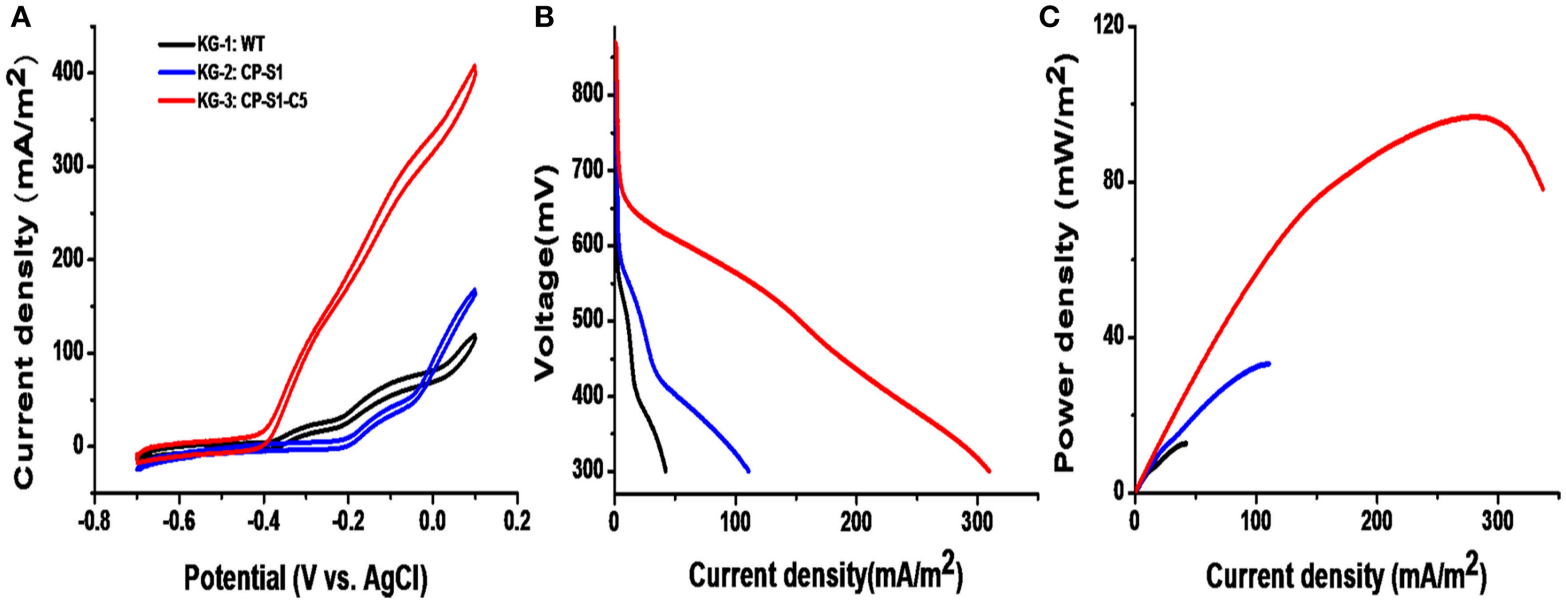
Electrochemical analysis of the performances of microbial consortia in the 20 mM glucose and 20 mM xylose-fed MFCs. **(A)** Turnover cyclic voltammetry (CV) curves at a scan rate of 1 mV/s. **(B)** MFC polarization curves obtained by linear sweep voltammetry (LSV) with a slow scan rate of 0.1 mV/s. **(C)** MFC power density output curves calculated on the basis of the corresponding polarization curves.

The cyclic voltammetry (CV) was conducted to reveal the redox reaction kinetics at the interfaces of bacterial cells and anodes with a scan rate of 1 mV/s (Li et al., 2018b,c). As shown in Figure 4A, there were typical redox peaks of favins in the CV curves starting from around ~-0.4 V (vs. Ag/AgCl), revealing that the dominating mechanism for bioelectricity production in this microbial consortium was the favins-mediated EET. Furthermore, another catalytic current arose at ~-0.27 V (vs. Ag/AgCl) was observed in these strains, which was caused by the direct involvement of conductive *c*-type cytochromes. To further investigate the MFCs' performance inoculated with these microbial consortia, the linear sweep voltammetry (LSV) and the polarization curves at a low scan rate of 0.1 mV/s were obtained (Baron et al., 2009). As shown in Figure 4B, the maximum catalytic current of KG-3: CP-S1-C5 was the highest (408.2 ± 8.0 mA/m^2^), which was higher than the maximum catalytic current of KG-1: WT (119.6 ± 6.0 mA/m^2^) and KG-2: CP-S1 (167.84 mA/m^2^). Notably, the dropping slope of the polarization curve obtained from the engineered KG-3: CP-S1-C5 was smaller than those obtained from the other two microbial consortium (KG-1: WT and KG-2: CP-S1), implying that the internal charge transfer resistance of the MFC inoculated with KG-3: CP-S1-C5 was relatively smaller (Figure 4C). The power densities were calculated for each; the engineered KG-3: CP-S1-C5 microbial consortium obtained a maximum power density of 104.7 ± 10.0 mW/m^2^, which was 7.2- and 2.2-folds higher than that of the KG-1:WT (12.7 ± 3.0 mW/m^2^) and KG-2:CP-S1 (32.7 ± 6.0 mW/m^2^), respectively.

**Figure 5:**
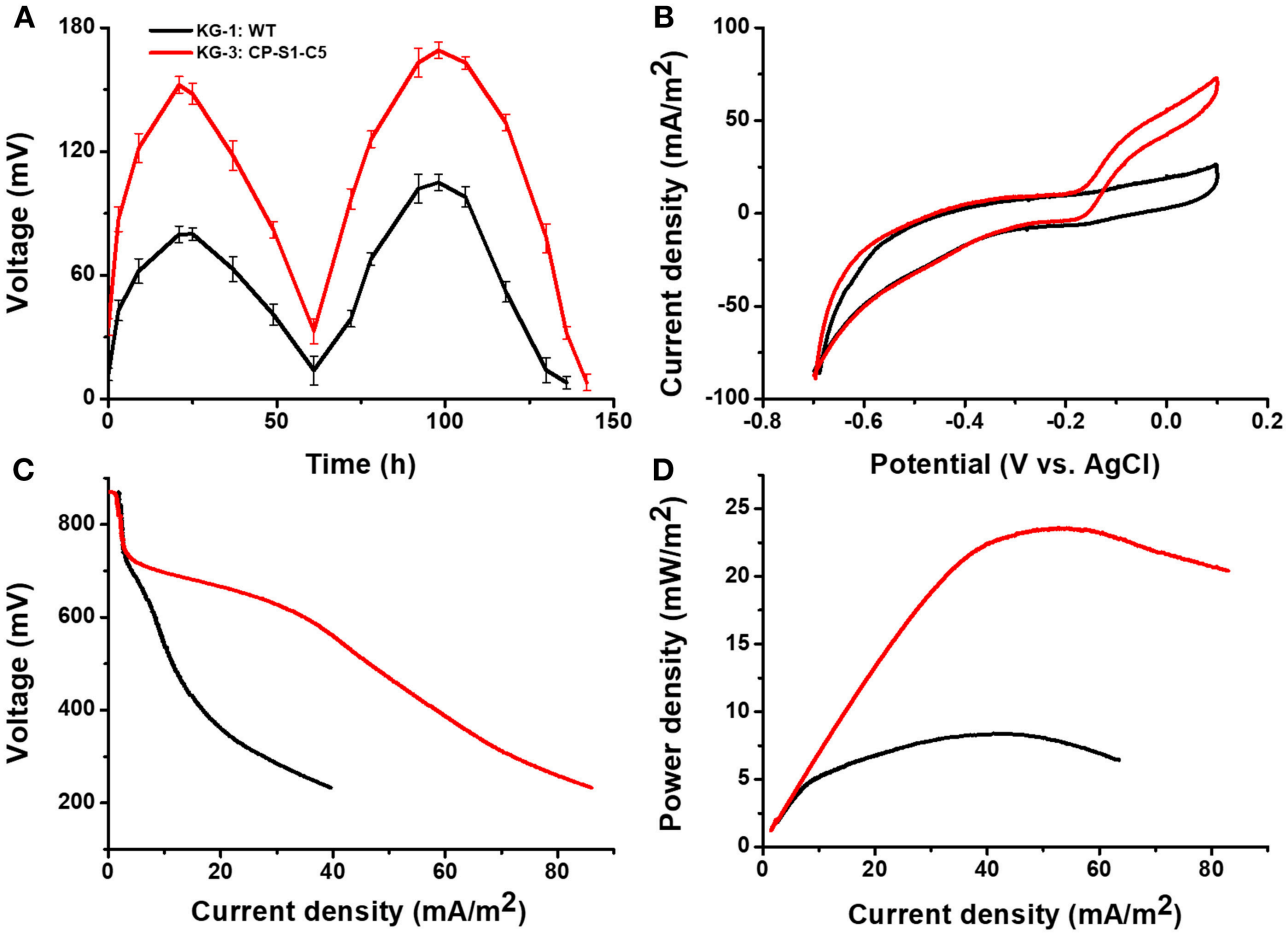
Electrochemical analysis of the performances of microbial consortia in the cellulose hydrolyzates-fed MFCs. MFC output voltage profiles produced by KG-3: CP-S1-C5 under cellulose hydrolyzates feedstock anolyte. **(A)** Voltage output in the multiple operational cycles. **(B)** Turnover cyclic voltammetry (CV) curves at a scan rate of 1 mV/s. **(C)** MFC polarization curves obtained by linear sweep voltammetry (LSV) with a slow scan rate of 0.1 mV/s. **(D)** MFC power density output curves calculated on the basis of the corresponding polarization curves.

To evaluate the electricity generation capacity of utilizing cellulose hydrolyzates, this synergistic microbial consortium (KG-3: CP-S1-C5) was further inoculated to the sterile or non-sterile corn straw hydrolyzates-fed MFC. As shown in Figure 5, the maximum output voltage of the sterile cellulosic hydrolyzates-fed MFCs could reach 152.3 ± 8.0 mV, the maximum current density was 72.8 ± 5.0 mA/m^2^, and the maximum power density of the sterile cellulosic hydrolyzates-fed MFC could reach 23.5 ± 6.0 mW/m^2^.

To further verify the stability of the engineered co-cultures, particularly over long term and in the presence of non-sterile feeds, we further used non-sterile corn straw hydrolyzates as the carbon source to evaluate the electricity generation capability. As shown in Figure S3, the output voltage of MFCs with a multi-cycle operation revealed that the engineered co-culture enabled a stable power generation. And the results of bioelectrochemical characterization also demonstrated that the engineered co-culture (KG-3: CP-S1-C5) showed a superior performance of electricity generation than that of co-culture (KG-1: WT). In all, our engineered co-culture showed stability of power generation in MFCs with either sterile or non-sterile feeds.

## Conclusions

To efficiently harvest electricity from glucose and xylose in cellulosic hydrolyzates, we rationally designed and constructed synergistic microbial consortia including *K. pneumoniae* and *S. oneidensis* in a hierarchical way, in which *K. pneumonia* could convert glucose and xylose into lactate to feed *S. oneidensis* continuously as the sole carbon source and electron donor.

To promote power generation of the microbial consortium in cellulosic hydrolyzates-fed MFCs, we rationally engineered the co-culturing strains in the consortium by redirecting carbon flux distribution toward lactate biosynthesis in *K. pneumoniae* and enhancing flavins-mediated EET efficiency of *S. oneidensis*, respectively. In this synthetic microbial consortium, the by-products (i.e., ethanol and acetate) were eliminated, and a lactate biosynthesis pathway was assembled into *K. pneumonia* for overproducing lactate. To facilitate extracellular electron transfer (EET) of exoelectrogens, a biosynthetic flavins pathway from *B. subtilis* was expressed in a highly hydrophobic *S. oneidensis* CP-S1, which improved both the direct-contacted and the flavins-mediated EET. Subsequently, the co-culturing conditions and substrate concentrations were further optimized to obtain higher and more stable electricity output. The glucose and xylose co-fed MFC inoculated with the recombinant *K. pneumonia S. oneidensis* generated a maximum power density of 104.7 ± 10.0 mW/m^2^. Furthermore, we used this synthetic microbial consortium in the corn straw hydrolyzates-fed MFC, obtaining a power density 23.5 ± 6.0 mW/m^2^. This rationally constructed a “fermenter-exoelectrogen” synthetic microbial consortium, that successfully utilized cellulosic hydrolyzates to harvest electricity, laying the foundation for the conversion of other biomass that was difficult to utilize in MFCs for power generation.

## Author Contributions

FL and XA designed the project, performed experiments, analyzed data, and drafted the manuscript. DW, JX, YuC, WL, YiC, XG, XL, CL, and SL helped to design some experiments and drafted the manuscript. HS designed and supervised the project, analyzed data, and critically revised the manuscript.

### Conflict of Interest Statement

The authors declare that the research was conducted in the absence of any commercial or financial relationships that could be construed as a potential conflict of interest.
