# Supplementary material for: Engineering Microbial Consortia for High-Performance Cellulosic Hydrolyzates-Fed Microbial Fuel Cells
Source: Front Microbiol. 2019 Mar 18;10:409. doi: 10.3389/fmicb.2019.00409 (PMC6432859; doi:10.3389/fmicb.2019.00409)
Supplement: Supplementary file 1 [file Data_Sheet_1.docx]

Supporting Information (SI)

**Engineering microbial consortia for high-performance cellulosic hydrolyzates-fed microbial fuel cells**

**Feng Li^1‡^, Xingjuan An^1‡^, Deguang Wu^2,3^, Jing Xu^1^, Yuanyuan Chen^1^, Wenchao Li^1^，Yingxiu Cao^1^, Xuewu Guo^3^, Xue Lin^4^, Congfa Li^4^, Sixin Liu^4^, Hao Song^1^***

^1^ Frontier Science Center for Synthetic Biology, Key Laboratory of Systems Bioengineering (MOE), School of Chemical Engineering and Technology, Tianjin University, Tianjin 300350, China

^2^ Department of Brewing Engineering, Moutai Institute, Renhuai 564500, Guizhou Province, China.

^3^ Tianjin Engineering Research Center of Microbial Metabolism and Fermentation Process Control, Tianjin University of Science and Technology, Tianjin 300457, China.

^4^ College of Food Science and Technology, Hainan University, Haikou 570228, Hainan Province, China.

^‡^ Equal contribution

* Corresponding author: H. Song, E-mail: hsong@tju.edu.cn

**Optimization of the seeding OD_600_ of *K. pneumoniae* and *S. oneidensis* in the glucose and xylose co-fed MFCs**

In order to maintain a stable MFC system inoculated with the K. pneumoniae and S. oneidensis co-culture, we optimize the initial co-cultivation of the two microorganisms by achieving a maximum voltage output via multimeter. We initially chosed 20 mM glucose and 20 mM xylose as substrate, four different proportions of seeding ratios were selected, such as OD_600 KG-3: CP-S1-C5_ =0.5:0.1; 0.5:0.5; 0.1:0.5; and 0.01:0.5. When the each microorganism grew up to OD_600_=2.0, the calculated cell suspensions were harvested by centrifugation with 5000 for 8 min at 4°C. Subsequently, we adjusted different OD_600_ of the seeding K. pneumoniae and S. oneidensis to obtain the optimal seeding ratio for maximized output voltage, when cell pellets were washed three times with M9 fresh buffer. The seeding OD_600_ ratio of 0.1:0.5 resulted in the hightest electricity ouput in MFCs (Figure S1).


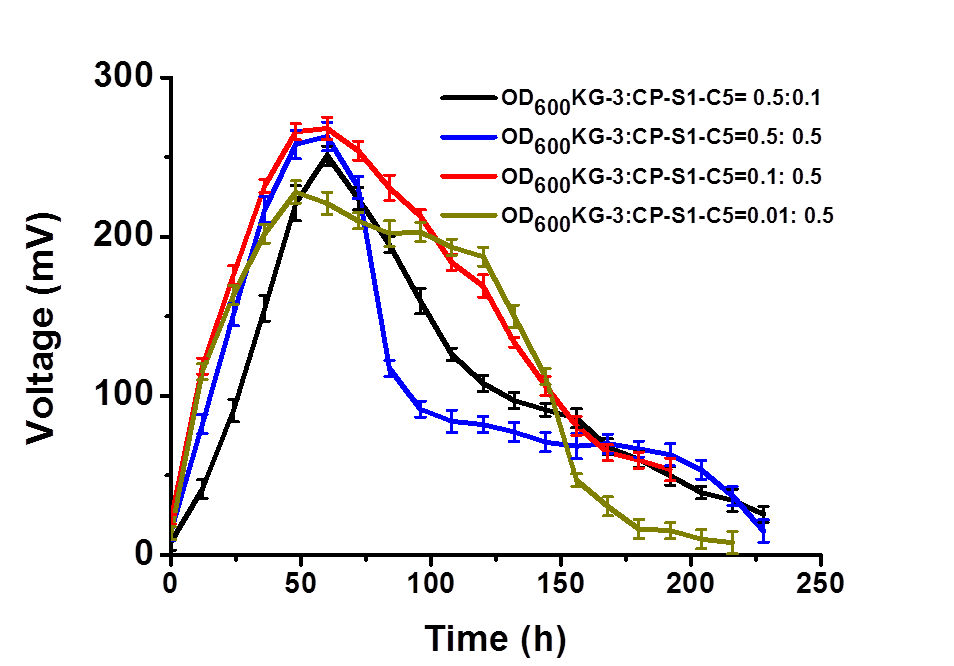


**Figure S1**. Optimization of the initial seeding ratio between *K. pneumoniae* and *S. oneidensis* in MFCs. With the substrate concentration were 20 mM glucose and 20 mM xylose, the output voltages of MFCs with different OD_600_ of seeding ratios were measured. Three independent replicates of MFC experiments were calculate

**Figure S2.** The proportion of each bacterium on the anode-attached biofilm of MFCs. Statistics were calculated from three independent replicates in this experiment.


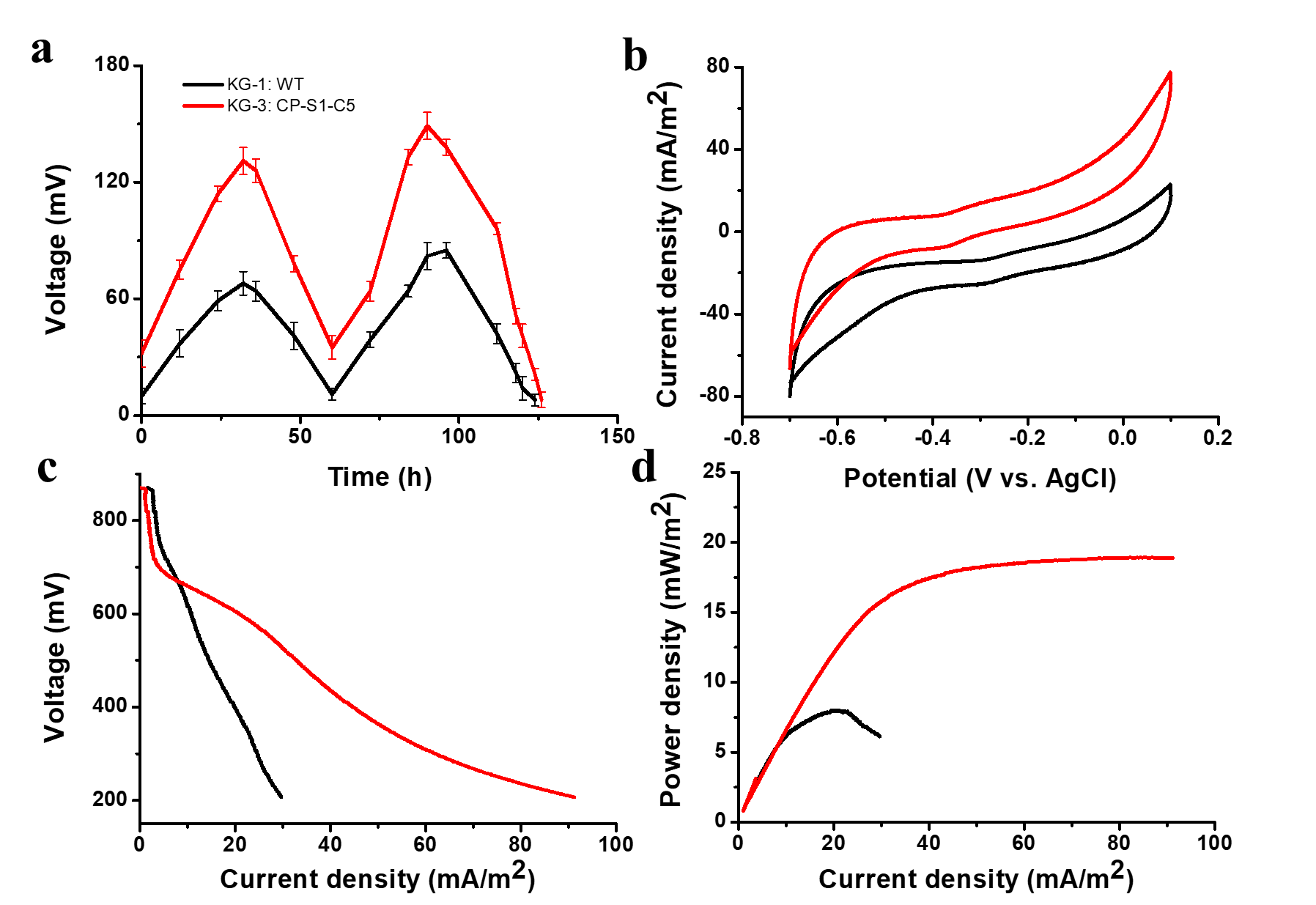


**Figure S3.** Electrochemical analyses of microbial consortia in non-sterile cellulose hydrolyzates-fed MFCs. (a) Time course of output voltage profiles produced by KG-3: CP-S1-C5 under non-sterile cellulose hydrolyzates feedstock anolyte. (b) Turnover cyclic voltammetry (CV) curves at a scan rate of 1 mV/s. (c) MFC polarization curves obtained by linear sweep voltammetry (LSV) with a slow scan rate of 0.1 mV/s. (d) MFC power density output curves calculated on the basis of the corresponding polarization curves.

**Table S1.** Original sequences of genes in this study

| *ldhD* (1002bp) | atgactaaaatttttgcttacgcaattcgtgaagatgaaaagccattcttgaaggaatgggaagacgctcacaaggacgtcgaagttgaatacactgacaagcttttgaccccagaaactgttgctttggcaaagggtgctgacggtgttgttgtttaccaacaacttgactacaccgctgaaactctgcaagctttggcagacaacggcatcactaagatgagcctgcgtaacgttggtgttgacaacatcgacatggctaaggctaaggaacttggcttccaaatcaccaacgttccagtttactcaccaaacgccatcgcagaacacgctgctatccaagctgcccgcatcctgcgtcaagacaaggctatggacgaaaaggttgcccgtcacgacttgcgttgggcaccaactatcggccgtgaagttcgcgaccaagttgttggtgttataggtactggccacatcggtcaagtcttcatgcaaatcatggaaggcttcggcgctaaggttatcgcttacgacatcttccgcaacccagaattggaaaagaagggctactacgtagactcacttgacgacctgtacaagcaagctgacgttatttccctgcacgttcctgacgttccagctaacgttcacatgatcaacgacgagtcaatcgctaaaatgaagcaagacgtagttatcgttaacgtatcacgtggtccattggttgacactgacgcggttatccgtggtttggactcaggcaagatcttcggttacgcaatggacgtttacgaaggtgaagttggcatcttcaacgaagactgggaaggcaaggaattcccagacgcacgtttagctgacttaatcgctcgtccaaacgttctggtaactccacacactgctttctacactactcacgctgttcgcaacatggtagttaaggccttcgacaacaaccttgaattggttgaaggcaaggaagctgaaactccagttaaggttggctaa |
| --- | --- |
| *lldP* (1656bp) | atgaatctctggcaacaaaactacgatcccgccgggaatatctggctttccagtctgatagcatcgcttcccatcctgtttttcttctttgcgctgattaagctcaaactgaaaggatacgtcgccgcctcgtggacggtggcaatcgcccttgccgtggctttgctgttctataaaatgccggtcgctaacgcgctggcctcggtggtttatggtttcttctacgggttgtggcccatcgcgtggatcattattgcagcggtgttcgtctataagatctcggtgaaaaccgggcagtttgacatcattcgctcgtctattctttcgataacccctgaccagcgtctgcaaatgctgatcgtcggtttctgtttcggcgcgttccttgaaggagccgcaggctttggcgcaccggtagcaattaccgccgcattgctggtcggcctgggttttaaaccgctgtacgccgccgggctgtgcctgattgttaacaccgcgccagtggcatttggtgcgatgggcattccaatcctggttgccggacaggtaacaggtatcgacagctttgagattggtcagatggtggggcggcagctaccgtttatgaccattatcgtgctgttctggatcatggcgattatggacggctggcgcggtatcaaagagacgtggcctgcggtcgtggttgcgggcggctcgtttgccatcgctcagtaccttagctctaacttcattgggccggagctgccggacattatctcttcgctggtatcactgctctgcctgacgctgttcctcaaacgctggcagccagtgcgtgtattccgttttggtgatttgggggcgtcacaggttgatatgacgctggcccacaccggttacactgcgggtcaggtgttacgtgcctggacaccgttcctgttcctgacagctaccgtaacactgtggagtatcccgccgtttaaagccctgttcgcatcgggtggcgcgctgtatgagtgggtgatcaatattccggtgccgtacctcgataaactggttgcccgtatgccgccagtggtcagcgaggctacagcctatgccgccgtgtttaagtttgactggttctctgccaccggcaccgccattctgtttgctgcactgctctcgattgtctggctgaagatgaaaccgtctgacgctatcagcaccttcggcagcacgctgaaagaactggctctgcccatctactccatcggtatggtgctggcattcgcctttatttcgaactattccggactgtcatcaacactggcgctggcactggcgcacaccggtcatgcattcaccttcttctcgccgttcctcggctggctgggggtattcctgaccgggtcggatacctcatctaacgccctgttcgccgcgctgcaagccaccgcagcacaacaaattggcgtctctgatctgttgctggttgccgccaataccaccggtggcgtcaccggtaagatgatctccccgcaatctatcgctatcgcctgtgcggcggtaggcctggtgggcaaagagtctgatttgttccgctttactgtcaaacacagcctgatcttcacctgtatagtgggcgtgatcaccacgcttcaggcttatgtcttaacgtggatgattccttaa |

**Table S2.** Main Ingredients of the M9 minimal medium

| Main Ingredients | Concentration |
| --- | --- |
| Na_2_HPO_4_ | 6 g/l |
| KH_2_PO_4_ | 3 g/l |
| NaCl | 0.5 g/l |
| NH_4_Cl | 1 g/l |
| MgSO_4_ | 1 mM |
| CaCl_2_ | 0.1 mM |

**Table S3.** Analyses of remaining substrate level at 24 hours under different feed-in concentrations of glucose and xylose

|  | KG-1: WT | | KG-2: CP-S1 | | KG-3: CP-S1-C5 | |
| --- | --- | --- | --- | --- | --- | --- |
|  | Glucose | Xylose | Glucose | Xylose | Glucose | Xylose |
| 10 mM glucose+  30 mM xylose | 7.2±1.2 | 19.3±0.8 | 3.4±0.4 | 10.7±0.9 | 0.9±0.3 | 6.1±0.9 |
| 20 mM glucose+  20 mM xylose | 12.1±0.4 | 14.8±1.1 | 6.2±0.8 | 8.4±1.2 | 1.5±0.7 | 4.8±0.4 |
| 30 mM glucose+  10 mM xylose | 18.3±0.7 | 8.1±0.5 | 9.3±1.3 | 5.3±0.7 | 2.1±1.0 | 2.4±0.8 |
